# Effects of dietary intervention in young female athletes with menstrual disorders

**DOI:** 10.1186/1550-2783-11-21

**Published:** 2014-05-26

**Authors:** Karolina Łagowska, Karina Kapczuk, Zbigniew Friebe, Joanna Bajerska

**Affiliations:** 1 Department of Human Nutrition and Hygiene, Dietetic Division, University of Life Sciences, Wojska Polskiego 28 str, 60-637 Poznań, Poland; 2 Department of Perinatology and Gynecology, Division of Gynecology, Karol Marcinkowski University of Medical Sciences in Poznań, Polna 33 str, 60-535 Poznań, Poland

**Keywords:** Menstrual disorders, Female athletes, Nutritional habits, Nutritional status, Dietary intervention

## Abstract

**Background:**

The aim of this study was to evaluate the influence of three months of dietary intervention on menstrual cycle in young female athletes with amenorrhea or oligomenorrhea.

**Methods:**

From forty-five female professional athletes with menstrual irregularity that were recruited thirty-one, aged 18.1 ± 2.6 years, completed the study and were analyzed. Hyperprolactinemia, thyroid dysfunction, primary ovarian failure and hyperandrogenism were excluded in the study participants. The subjects started intense training at the age of 11.2 ± 3.5 years and continued during next 6.8 ± 3.3 years. Energy and nutrients intake, total energy expenditure, energy availability and body composition as well as serum concentrations of LH, FSH, 17 – beta estradiol and progesterone were measured at the beginning of the study and after three months of individualized dietary intervention.

**Results:**

Following three months of dietary intervention significant increase in energy intake (2354 ± 539 vs. 258 8 ± 557 kcal, P = 0.004) and energy availability (28.3 ± 9.2 vs. 35.8 ± 12.3 kcal/kg FFM/d, P = 0.011) was observed as well as improved energy balance (−288 ± 477 vs. -51 ± 224 kcal/d, P = 0.002). Though no changes in BMI and body composition were noted but significant rise in LH concentrations (3.04 ± 1.63 vs. 4.59 ± 2.53 mIU/ml, P = 0.009) and LH to FSH ratio (0.84 ± 0.56 vs. 0.96 ± 0.52, P = 0.001) was achieved, but no restoration of menstrual cyclicity.

**Conclusions:**

This report provides further support for the role of energy deficiency in menstrual disorders among young female athletes and the benefits of an adequate energy intake and energy availability on hormones concentration. Continuation controlled dietary intervention is needed to assess the extent to which long-term improvement in the nutritional status results in improvements in the hormonal status of female athletes, to an extent that would allow the regulation of the menstrual cyclity.

## Background

The strenuous physical activity of professional female athletes may generate serious health problems. It is estimated that between 16% [1] and 61% [2] of female athletes suffer from hypothalamic-pituitary menstrual disorders. In studies conducted by Torstveit et al. [3], the frequency of menstrual disorders among elite female athletes was 34.5% in aesthetic disciplines, 30.9% in endurance disciplines, 23.5% in weight class disciplines, 17.6% in anti-gravitation disciplines, 16.7% in technical disciplines, 12.8% in ball game and power sport disciplines.

There is a disturbingly low level of knowledge among athletes of different sports disciplines regarding the potential health effects of untreated menstrual dysfunctions [4,5]. Young female athletes are not aware that a long-term negative energy balance, inadequate nutrient intake, and endocrine disorders including the hypothalamic-pituitary-ovarian axis are particularly dangerous in the period of achieving the peak bone mass and may contribute to metabolism disturbances in the skeletal tissue. Christo et al. [6] observed significantly lower BMD values in the lumbar spine area among athletes with menstrual disorders compared to physically active and sedentary women with regular cycles. The study of Nicolas et al. [7] also showed a significantly decreased bone density in athletes suffering from amenorrhea and oligomenorrhea. Studies of athletes with amenorrhea and low bone mass showed that even after the restoration of the menstrual cycle bone density remained significantly lower compared to the average value of women in this age group [8].

Prolonged menstrual disorders have a negative effect on the quality and quantity of plasma lipoproteins, which favors the formation of atherosclerotic lesions. Significant differences in blood lipid parameters in athletes with amenorrhea compared to athletes with regular cycles have been demonstrated. In the study of Rickenlund et al. [9], athletes with amenorrhea had significantly higher levels of total and LDL cholesterol compared to athletes and sedentary women with regular cycles. The increase in the LDL levels was higher when the energy intake was lower.

Taking the afore mentioned into account it seemed appropriate to take steps to limit menstrual disorders and their negative health effects. The aim of this study was to evaluate nonpharmacological dietary interventions on the menstrual disorders in young female athletes.

## Methods

### Subjects

Forty-five well-trained female athletes with menstrual disorders (18 rowers, 12 synchronized swimmers, 15 triathlonists) were recruited from different sports club in Poznań and thirty-one the (12 rowers, 8 synchronized swimmers, 11 triathlonists) completed a dietary intervention. The inclusion criteria were: menstrual irregularity within the last 12 months, a training period of at least 3 years, training session > 4/wk, no serious medical conditions, no use of hormonal contraception or other medications that might interfere with the hypothalamic-pituitary-gonadal axis activity, no clinical diagnosis of an eating disorders, no history of clinical diagnosis of primary ovarian failure, hyperprolactinemia, thyroid dysfunction or polycystic ovary syndrome and non-smoking.

Written informed consent was obtained from all participants or their parents. The study was approved by the Poznań Medical Ethics Committee (no. 334/09).

### Menstrual status

Each subject completed a two-part medical questionnaire. The questions in the first part concerned menstruation: age at menarche, length of the menstrual cycles, and history of amenorrhea. Part two of the questionnaire referred to sport activities: age at the beginning of training, training period, number of training session per week, hours of training per day and per week.

Primary amenorrhea was diagnosed where there was no onset of menses by 15 years, while secondary amenorrhea was diagnosed when there was no menstruation for 6 months, or for more than three times the previous cycle length. Menstrual periods that occurred more than 35 days apart were described as oligomenorrhea [10].

Each participant underwent gynecological evaluation, including a pelvic ultrasound and measurements of luteinizing hormone (LH), follicle-stimulating hormone (FSH), progesterone (P), 17β - estradiol (E2), prolactin (PRL), thyroid-stimulating hormone (TSH), testosterone (T), and sex-hormone-binding globulin (SHBG) serum concentration, in order to exclude independent causes of amenorrhea or oligomenorrhea (such as pregnancy, primary ovarian failure, hyperprolactinemia, thyroid dysfunction or polycystic ovary syndrome).

### Blood sampling and biochemical analyses

Blood samples were obtained in menstruating subjects between days 2 and 5 of the menstrual cycle (in the early follicular phase), and at random in amenorrheic subjects. Blood serum samples were taken between 6.00 a.m. and 9.00 a.m. following overnight fasting and rest. The athletes were instructed to abstain from caffeine and alcohol for 24 hours prior to the blood sampling, and to refrain from performing strenuous exercise on the day of sampling.

Serum concentration of LH, FSH, E2, P, PRL, TSH, T and SHBG were measured by immunochemical methods using Chemiluminescent Microparticle Immunoassay (CMIA) and Microparticle Chemiflex Flexible interassay protocols and making use of diagnostic sets and an ARCHITECT automatic analyzer. Serum leptin levels were estimated using Human Leptin Elisa by LINCO Research. All hormones concentrations were determined in duplicated.

### Body weight and body composition measurements

In order to evaluate the nutritional status, the anthropometrical indices, height and weight were measured using an anthropometer coupled with a WPT 200 OC verified medical scale (Rad Wag). BMI (kg/m^2^) was calculated as body weight divided by squared body height. The participants were dressed in minimal clothing during the measurements, which were rounded to the nearest 0.5 kg and 0.5 cm. Analysis of body fat mass (FM) and fat-free mass (FFM) was performed in the morning, following an overnight fast, with the subjects lying in a supine position, using BODYSTAT 1500, as described by Heyward et al. [11].

### Resting metabolic rate

Resting metabolic rate (RMR) was assessed by using a portable indirect calorimeter for 25 minutes (Cosmed K4b2, Cosmed, Italy). A face mask (Hans Rudolph, Kansas City, MO) covering the mouth and nose of the participant was attached to a bidirectional digital turbine flow-meter and fastened to the participant using a mesh hairnet with Velcro straps. To guarantee an airtight seal, a disposable gel seal (Hans Rudolph) was positioned between the inside of the face mask and the skin. The Cosmed K4b2 system was calibrated prior to each individual test according to the manufacturer’s guidelines. Breath-by-breath O_2_ and CO_2_ gas exchange was measured and recorded in the portable unit’s computer system. On completion of each test, the stored data were transferred to the Cosmed K4b2 version 6 computer software running on a Windows-based laptop computer. The data were then averaged over 15 second intervals and transferred to Microsoft *Excel* for further analysis. The morning before the RMR measurements, the Cosmed K4b2 was calibrated with a calibration gas mixture (16% O_2_, 5% CO_2_). The test was carried out with the participant in a comfortable supine position, at an environmental temperature of 21–22°C. All measurements were done in the morning (between 6 and 9 a.m.) following a 12 hours fast and a minimum of 8 hours of rest. The results of the RMR measurement were compared with the RMR predicted by the Harris-Benedict equation [12] and the RMR(kcal)/FFM(kg) ratio was also calculated.

### Energy and nutrients intake

Seven consecutive days of dietary records were obtained under the supervision of dieticians. Athletes had a regularly contact with registered dietitian who teach them and control how to record nutrition intake. All meals (including recipes and item masses), nonmeal foods, beverages, and fluids were recorded in diary form using a photographic album of dishes [13]. The daily diets were analyzed for their energy and nutrient levels (fat, protein, carbohydrate, dietary fiber, calcium, phosphorus, iron, zinc, vitamins A, D, B_1_, B_2_, niacin, B_6_, B_12_, foliate and vitamin C) using the *Dietician* computer software package, based on Polish food composition tables [14].

### Total energy expenditure and energy availability

For three days, each subject wore a heart-rate monitor (HR) (Polar Sport Tester, RS 400, Finland) in order to estimate total energy expenditure (TEE). For each subject, the relationship between HR and VO_2_ was established. The measurements were carried out two or more hours after meals, and after the subject had rested for 30 min, having arriving at the laboratory. Results were obtained by simultaneous measurement of HR and VO_2_ for the following activities carried out sequentially: lying in supine position, sitting quietly, standing quietly, and continuous graded exercise on a cycle ergometer. After preliminary editing to remove spurious HR data, the total energy expenditure (TEE) was calculated using the Flex-HR method. This method requires the definition of a Flex-HR for each subject, above which there is a good correlation between HR and VO_2_, but below which there is a poor correspondence between the two parameters. The Flex-HR was calculated as the mean of the highest HR for the resting activities (supine, sitting, and standing) and the lowest HR of the exercise activities. At the end of the measurement session, researchers transferred the minute-by-minute records of the last twenty-four hours from the instrument to a database. The 24-hour energy balance (EB) was calculated as the difference between the means of seven consecutive days of 24-hour energy intake and the TEE as a mean of three days. Energy availability (EA) was calculated by subtracting exercise energy expenditure (EEE) from total daily energy intake, and was adjusted for FFM kg [10].

### Dietary intervention

After the evaluation of the participants’ nutritional habits, all the athletes were informed of nutritional mistakes in their current diets and of the health consequences of dietary deficiencies. Then, for each of the athletes who was qualified for the study, we prepared an individual diet. Taking into account the energy balance and the energy availability, the daily energy intake was established on the basis of the individual energy requirements that had been calculated from the total energy expenditure data. The recommended level of protein intake was determined in accordance with the recommendations of the American College of Sports Medicine Female Athlete Triad Position Stand (ACSM) [10], taking into account 1.2–1.6 g/kg/d intake. Using the recommendations of Manore et al. [15], the level of carbohydrates and fat intake was determined, which respectively amounted to a minimum of 55% and 25–30% of the daily energy intake. Adequate daily intake for calcium (1000–1300 mg) and vitamin D (400–800 IU or 10–20 mcg) are based on the ACSM recommendations [10] and on Roupas et al. [16] results. The recommended intake of other vitamins and minerals was established in accordance with Recommended Dietary Allowances for girls aged 16–18 years and women over 19 years, in accordance with Jarosz et al. [17]. The dietary counseling session also included a discussion of special foods for athletes, sports drink, supplements, shopping tips, low-fat and low-calorie food, food preparation, dining out, iron, calcium and vitamins in foods. After first and second month of nonpharmacological dietary intervention, the control of following dietary intervention was conducted. Repeated assessments of total energy expenditure (1 day), energy availability, and the energy and nutrient values of daily diets (3 days) were conducted (data no shown). After third month the control of effect of dietary intervention was conducted and then total energy expenditure (3 day), energy availability, and the energy and nutrient values of the athlete’s daily diets (7 days), LH, FSH, E2 and P serum concentration were repeated by measured. To statistical analysis we used data from baseline of study and after third month of dietary intervention.

### Statistical analysis

Means and standard deviations of the quantitative variables were calculated. The normality of the distribution was checked. Comparisons between data from before and after the three-month dietary intervention were carried out using a *t*-test for independent variables. Connection between energy availability and LH serum concentration were carried out using Spearman’s rank correlation test. Statistical analyses were performed using *Statistica 8.0* software (StatSoft, 2008). *P*-values of less than 0.05 were considered statistically significant.

## Results

### Subjects characteristic

The subject characteristics of those who completed the study are shown in Table 1. The investigated group consisted of 5 secondary amenorrheic subjects and 26 oligomenorrheic subjects.

**Table 1:** Baseline group characteristics M ± SD

**Parameters**	
**Baseline characteristics**	
Age (years)	18.1 ± 2.6
Age at menarche (years)	13.0 ± 1.2
Age at the beginning of training (years)	11.2 ± 3.5
Training period (years)	6.8 ± 3.3
Number of training session per week (n/d)	5.2 ± 1.1
Hours of training per day (hours/d)	4.0 ± 1.8
Hours of training per week (hours/wk)	19.5 ± 7.2
RMR predicted (kcal/d)	1458 ± 56
RMR measured (kcal/d)	1354 ± 151
RMR measured/predicted*100%	92.8 ± 10.0
RMR measured - RMR predicted (kcal/d)	−105.0 ± 146.8
RMR/FFM (kcal/kg)	29.0 ± 3.6
**Hormonal parameters**	
TSH (0.35 –4.94 μIU/ml)	1.74 ± 0.80 (0.74–4.37)
PRL (5.18–26.53 ng/ml)	13.0 ± 9.33 (3.71–50.5)
T (10–90 ng/dl)	37.28 ± 21.85 (0.15–90.0)
SHBG (19.80–155.20 nmol/l)	62.79 ± 41.91 (18.0–228.4)

### Effect of the three month dietary intervention on energy and nutrient intake, energy balance, energy availability, body weight and composition

Three months of dietary intervention changed dietary habits of the study participants and resulted in significant increase in energy (mean 234 kcal/d), protein (mean 8 g/d), carbohydrate (mean 66.8 g/d), calcium (mean 146 mg/d), magnesium (mean 56 mg/d), vitamin A (450.9 mg/d), vitamin D (0.67 μg/d), foliate (mean 49.2 μg/d) and vitamin C (mean 53.9 mg/d) intake. EB and EA before and after the intervention differed significantly in the study subjects (mean 237 kcal/d and 7.5 kcal/kg FFM/d, respectively) (Table 2). No significant changes in athletes’ body weight, BMI and body composition were observed (Table 3).

**Table 2:** Energy and nutrients intake at 0 and 3 measurement points M ± SD

**Energy and nutrients**	**0**	**3**	p – value*
Energy (kcal)	2354 ± 539	2588 ± 557	0.041
Fat (g)	92.2 ± 27.5	84.2 ± 20.4	NS
Protein (g)	75.6 ± 14.8	85.5 ± 15.6	0.004
Carbohydrate (g)	305.4 ± 78.0	372.2 ± 86.3	< 0.001
Dietary fiber (g)	20.1 ± 5.4	21.8 ± 5.4	NS
Calcium (mg)	816.3 ± 232.9	963.3 ± 247.5	0.021
Phosphors (mg)	1442.0 ± 333.9	1435.1 ± 327.4	NS
Iron (mg)	11.1 ± 3.3	12.8 ± 3.2	NS
Zink (μg)	10.1 ± 3.0	11.0 ± 2.8	NS
Magnesium (mg)	275.0 ± 87.5	331.0 ± 80.7	0.003
Vitamin A (μg)	645.0 ± 234.8	1095.9 ± 655.1	< 0.001
Vitamin D (μg)	2.34 ± 1.42	3.01 ± 1.04	0.040
Vitamin E (mg)	9.9 ± 4.2	9.2 ± 3.4	NS
Vitamin B_1_ (mg)	1.20 ± 0.56	1.28 ± 0.26	NS
Vitamin B_2_ (mg)	1.80 ± 0.50	1.72 ± 0.46	NS
Niacin (mg)	12.5 ± 4.1	14.3 ± 3.3	NS
Vitamin B_6_ (mg)	1.80 ± 0.73	2.35 ± 0.94	NS
Foliate (μg)	202.7 ± 62.4	251.9 ± 64.4	0.014
Vitamin B_12_ (μg)	2.78 ± 1.47	3.67 ± 1.61	NS
Vitamin C (mg)	57.3 ± 24.4	111.2 ± 87.1	0.002
TEE (kcal/d)	2642 ± 348	2638 ± 421	NS
EB (kcal/d)	−288 ± 477	−51 ± 224	0.002
EEE (kcal/d)	959 ± 174	905 ± 337	NS
EA (kcal/kg FFM/d)	28.3 ± 9.2	35.8 ± 12.3	0.011

*Before dietary intervention (0) vs. after three months of dietary intervention (3).

**Table 3:** Anthropometric characteristics at 0 and 3 measurement points M ± SD

**Parameters**	**0**	**3**	p-value*
Body weight (kg)	59.3 ± 5.3	59.6 ± 5.3	NS
BMI (kg/m^2^)	20.6 ± 1.4	20.7 ± 1.5	NS
FM (%)	20.6 ± 3.7	21.0 ± 3.5	NS
FM (kg)	12.2 ± 2.4	12.5 ± 2.4	NS
FFM (%)	79.4 ± 3.7	79.0 ± 3.7	NS
FFM (kg)	47.1 ± 4.9	47.1 ± 4.8	NS

*Before nutritional intervention (0) vs. after three months of dietary intervention (3).

### Effect of the dietary intervention on hormonal parameters

**Figure 1:**
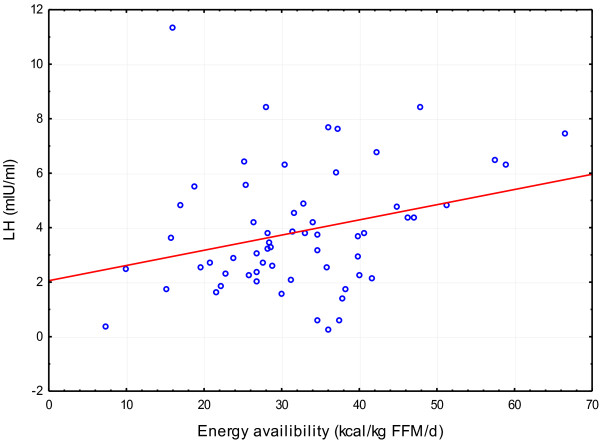
Correlation between energy availability and LH levels.

Neither resumption of regular cycles nor improved menstrual frequency was observed in the athletes during the three month study period. However, LH concentration and LH to FSH ratio measured after three months of dietary intervention were found to be significantly higher than at the beginning of the study (mean 41.55 mlU/ml and 0.12, respectively) (Table 4). A positive correlation between EA and LH concentrations appeared (r = 0.26, p < 0.05) (Figure 1).

**Table 4:** Hormones concentration at 0 and 3 measurement points M ± SD

**Hormones (reference values)**	**0**	**3**	p-value*
LH (2.39–6.60 mlU/ml)	3.04 ± 1.63	4.59 ± 2.53	0.009
FSH (3.03–8.08 mlU/ml)	5.01 ± 2.37	5.00 ± 2.08	NS
E2 (21–251 pg/ml)	36.5 ± 19.4	36.2 ± 15.3	NS
P (0.1–0.3 ng/ml)	0.54 ± 0.99	0.68 ± 0.77	NS
LH/FSH (0.6–1.2)	0.84 ± 0.56	0.96 ± 0.52	0.001

*Before dietary intervention (0) vs. after three months of dietary intervention (3).

## Discussion

In the study, the authors evaluated the effects of an individualized dietary intervention, providing an appropriate energy availability, energy balance and an adequate intake of minerals and vitamins, on the menstrual cycle in young female athletes. Diets were planned by taking into account the total energy expenditure, nutritional status and the current training period, in the expectation that an individualized diet will help reduce menstrual dysfunctions without decreasing total energy expenditure, training volume and hormonal treatment. The planned study period was nine months, and this study provides results obtained after three months, the first time-point, post dietary intervention start.

Our results concerning energy and nutritional intakes, obtained before the start of the above dietary intervention, were similar to our previous results [18,19]. They were also comparable to those obtained by Hoch et al. [20] and Tomten et al. [21], who also demonstrated energy availability below 30 kcal/kg FFM/d and the negative energy balance in athletes with menstrual disorders. Furthermore, similarly to studies by Manore [15], Hoogenboom et al. [22], Quah et al. [23] and Woolf et al. [24], daily diet values for most vitamins and minerals indicated deficiency. In study participants, the RMR was also lower than predicted value. Similar to Mallinson et al. [25], we used the RMR/pRMR ratio as an indicator of the energy status. The mean value obtained was 92.8 with a range of 72.3-115.5, potentially indicating an energy deficiency in some part of study participants. Many authors suggested that body weight alone and an intensive physical activity are not sufficient to explain the onset of menstrual disorders. Many authors suggested that menstrual dysfunction occurs only in the presence of relative caloric deficiency resulting from inadequate nutritional intake precluding achievement of an appropriate energy expenditure. They also emphasize that this is the most important factor leading to menstrual disorders development [26,27]. Results presented by Thong et al. [28] also showed an inadequate energy intake among female athletes with amenorrhea. In the above case, energy availability was 50% lower compared to regularly menstruating women (16 kcal/d/kg FFM and 30 kcal/d/kg FFM, respectively). The relationship between normal functioning of the hypothalamic-pituitary-gonadal axis and an adequate energy intake under stress conditions was already demonstrated in the 1980s. In runners, Kaiserrauer et al. [29] showed that the use of a low-energy diet, deficient in protein and fat, may contribute to progesterone serum concentrations reduction and the luteal phase shortening.

In athletes’ daily diets, the control of energy and nutrients intakes demonstrate significant variations. Despite mean values showing an increase of energy and nutrients intakes, the high standard deviation indicates that not all study participants adhere to the recommendations of the dietary intervention. This situation demonstrates how difficult it is to implement an individual diet in this group of subjects. During a three-month dietary intervention, an increased energy availability in the studied athletes was also observed. Additionally, the energy availability exceeded the critical value of 30 kcal/kg FFM/d. In athletes with menstrual disorders, Nattiv et al. [10] and DeSouza [30] indicated that an increased energy availability, and not the weight gain alone, is the most important factor for the restoration of regular menstrual cycles. Loucks et al. [25] suggested that the pulsatile secretion of LH depends on the energy availability, which was also confirmed in this paper (significant relationship between LH and energy availability). In our paper, despite the fact that after three months of non-pharmacological dietary intervention none of study participants resumed regular menses, LH and LH to FSH ratio significantly increased. In athletes, a surprising lack of changes in body weight and composition may be explained by decreased level of baseline RMR resulting from the long-term energy deficiency. Moreover, diets implemented during this dietary intervention aimed to provide a sustainable energy balance, thus to avoid weight gain.

In athletes, Dueck et al. [31] and Kopp-Woodroffe et al. [32] demonstrated the resumption of menses after approximately 6 months and 9–12 weeks, respectively. Competitive athletes should be counseled that the sustained resumption of menses (involving regular menstrual cycles of 36 days or less occurring in the period of 3 months or more) may take longer than one year, when non-pharmacological therapy is implemented. Arends et al. [33] found that the restoration of regular menstrual cycles in female athletes is possible after increasing the energy value of daily meals contributing to body weight and BMI increase. In the group of 373 female athletes, after five-year non-pharmacological dietary therapy, regular menstrual periods returned in 17.6% subjects. Moreover, in this group, a significant increase in BMI, from 20.8 ± 0.5 kg/m^2^ to 22.7 ± 0.6 kg/m^2^ (p < 0.005), as well as in body weight, from 58.0 ± 2.0 kg to 63.3 ± 2.3 kg (p < 0.005), were also observed. However, no information on body composition of the athletes from the above group were obtained. Dueck et al. [31] showed LH pulsatility accompanied by the weight gain of approximately 3 kg and a 6% body fat increase. In contrast, Loucks et al. [34,35] have suggested that body weight changes are not associated with menstrual disturbances in athletes, probably due to adaptive energy-conserving mechanisms development allowing for the maintenance of body weight despite poor energy availability. Mallinson et al. [25] compared and contrasted responses of two exercising women with amenorrhea of varying duration to an intervention of increased energy intake. This study was very similar to ours due to implementation of a non-pharmacological dietary intervention without reducing the energy expenditure or the intensity and volume of training. In the case study conducted by Mallinson et al. [25], resumption of menses occurred 23 and 74 days into the intervention for the women with short-term and long-term amenorrhea, respectively. Recovery of regular menses and onset of ovulation coincided closely with increases in energy intake, weight gain and improvements in the metabolic environment.

In female athletes, difficulties in the restoration of regular menstrual cycles may result from multiple overlapping causes of such disorders. Bruni et al. [36] reported that inadequate dietary habits, extensive physical activity and stress are key factors differentiating women with menstrual disorders. The above situation demonstrates that an eventual use of a homogenous treatment in the population of women with menstrual disturbances or amenorrhea is impossible. Potential factors affecting menstrual cycle include various genetic, neuroendocrine and metabolic aspects. It seems that in the specific population included in our studies, all above mentioned factors, predisposing to such disorders, are present. Nattiv et al. [10] and Manore et al. [15] emphasized that an appropriately balanced diet with reduced training volume and intensity is the only possible way to alleviate menstrual disorders in female athletes. The present study is valuable because it is based on an individual, non-pharmacological diet intervention taking into account everyday burden of an intense physical effort without reduction of intensity and volume of everyday activities, which could be, according to authors’ knowledge, a potential cause of subject’s withdrawal from the study. In case of female athletes aiming to achieve desired results, the limitation of training sessions intensity is potentially difficult to accept intervention, therefore it was not suggested to study participants.

This study has several limitations. Firstly, LH and FSH concentrations were assessed only once before the start of dietary intervention, and then after three months. We did not determinate the pulsatile nature of those hormones, thus an assessment of the presence of ovulatory cycles in menstruating women was impossible. Secondly, the body composition was determined using the electrical bioimpedance method, which potentially raises some controversies. However, DEXA method was not used due to young age of study participants, tests frequency, and potential adverse (UV) effects.

## Conclusion

This report provides further support for the role of energy deficiency in menstrual disorders among young female athletes and the benefits of an adequate energy intake and energy availability on hormones concentration. Continuation controlled dietary intervention is needed to assess the extent to which long-term improvement in the nutritional status results in improvements in the hormonal status of female athletes, to an extent that would allow the regulation of the menstrual cyclity.

## Competing interests

The authors declare that they have no competing interests.

## Authors’ contributions

KŁ (corresponding author) was responsible for the study design, the statistical analysis, execution of the measurements and the writing of the manuscript. KK was involved in the execution of the measurements and the writing of the manuscript. ZF provided assistance in the study design and JB provided assistance in the editing of the manuscript. All authors read and approved the final manuscript.
